# Supplementary material for: The Plasma Membrane P-Type ATPase CtpA Is Required for Mycobacterium tuberculosis Virulence in Copper-Activated Macrophages in a Mouse Model of Progressive Tuberculosis
Source: Biomedicines. 2025 Feb 11;13(2):439. doi: 10.3390/biomedicines13020439 (PMC11853030; doi:10.3390/biomedicines13020439)
Supplement: Supplementary file 1 [file biomedicines-13-00439-s001.zip › biomedicines-3392823-supplementary.pdf]

Supplementary Materials:

Table S1. Bacterial strains, plasmids and primers used in this study.

Strains	Relevant Features	Reference
Mycobacterium tuberculosis		
H37Rv	Slow-growing attenuated strain, Amp^R, Chx^R, Cb^R	ATCC 25618
H37Rv:pJV53	Recombineering strain (with pJV53), Amp^R, Chx^R, Cb^R, Km^R	[11]
H37RvΔ*ctpA*	Δ*ctpA*, gene replaced by a Hyg^R cassette	This study
**Plasmids**	**Relevant features**	**Reference**
pJV53	Derivative of pLAM12 with Che9c 60–61 genes under control of the acetamidase promoter	Gift from Unizar
pYUB854	Hyg^R cassette is flanked by the γδ-res sites and by two MCSs for directional cloning of the homologous recombination substrates	Gift from Unizar
pALT25	549 bp upstream and 558 bp downstream of *Mtb Rv0092* (*ctpA*) in pYUB854	[21]

Table S2. qPCR-Primer sequences used in this study.

mRNA	Sequence 5′→3′	Tm (°C)	Product Size (bp)	Reference
*gapdh*_ Dir	CATTGTGGAAGGGCTCATGA	61	187	[39]
*gapdh*_ Rev	GGAAGGCCATGCCAGTGAGC	61		
*IL-1β*_ Dir	GCCACCTTTTTGACAGTGATGAG	62	99	(INCMNSZ)
*IL-1β*_ Rev	GACAGCCCAGGTCAAAGGTT	60		
Nos2 _Dir	CAAGCACATTTGGGAATGGAGA	60	141	[37]
*Nos2*_Rev	CAGAACTGAGGGTACATGCTGGAG	60		
Nox2 _Dir	GTGCACCATGATGAGGAGAA	60	97	[38]
Nox2 _Rev	TTGCAATGGTCTTGAACTCG	60		

Table S3. Efficiency and programs for relative quantification by RT-qPCR of genes expressed by *Mtb*-infected MH-S cells.

gen	Amplification Program (qPCR)	Th	Efficiency	Reference
gapdh	95ºC *x*5min + [95°C *x*10s + 61.2°C *x*10s + 72°C *x*15s] *x*39	5.66	1.99	This Study
IL-1β	95°C *x*5min + [95ºC *x*5s + 60°C *x*30s +72°C *x*5s] *x*40	51.24	2.09	This study
Nos2	95°C *x*5min + [95°C x5s + 60°C x30s +72°C x5s] *x*40	44.42	2.08	This Study
Nox2	95°C *x*5min + [95°C *x*5s + 59°C *x*20s + 72°C *x*30s] *x*40	62.22	2.07	This study

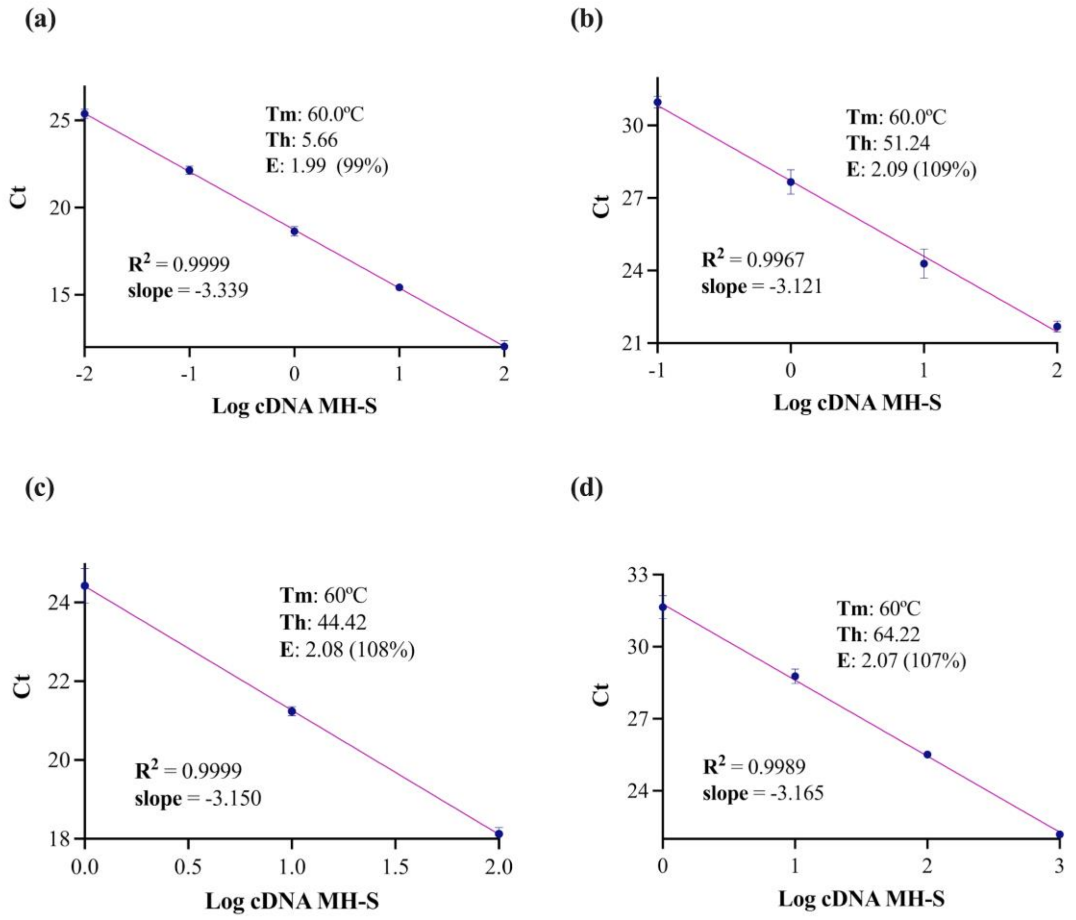

Figure S1. Oligonucleotide pair qPCR Amplification Efficiency Curves (**a**) *gapdh*_Dir and *gapdh*_Rev (**b**) *IL-1β*_Dir and *IL-1β*_Rev (**c**) *Nos2*_Dir and *Nos2*_Rev (**d**) *Nox2*_Dir and *Nox2*_Rev.

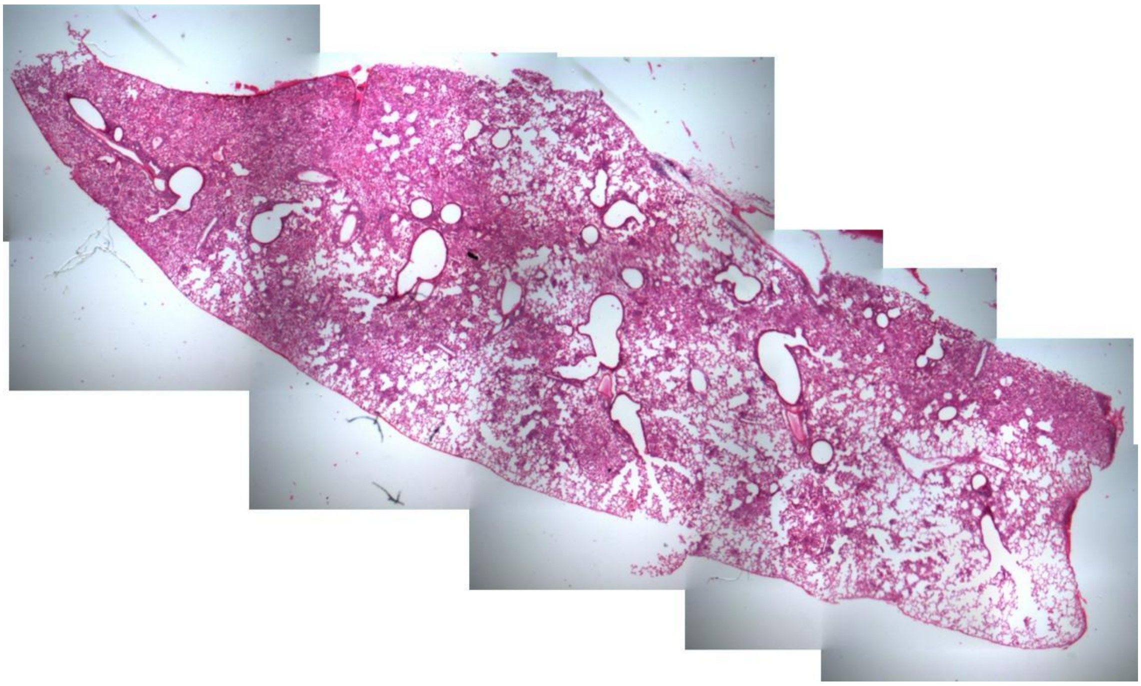

Figure S2. Hematoxylin and eosin (H&E) stained lung tissues BALB/c mice infected with *Mtb*H37Rv at 60 dpi.

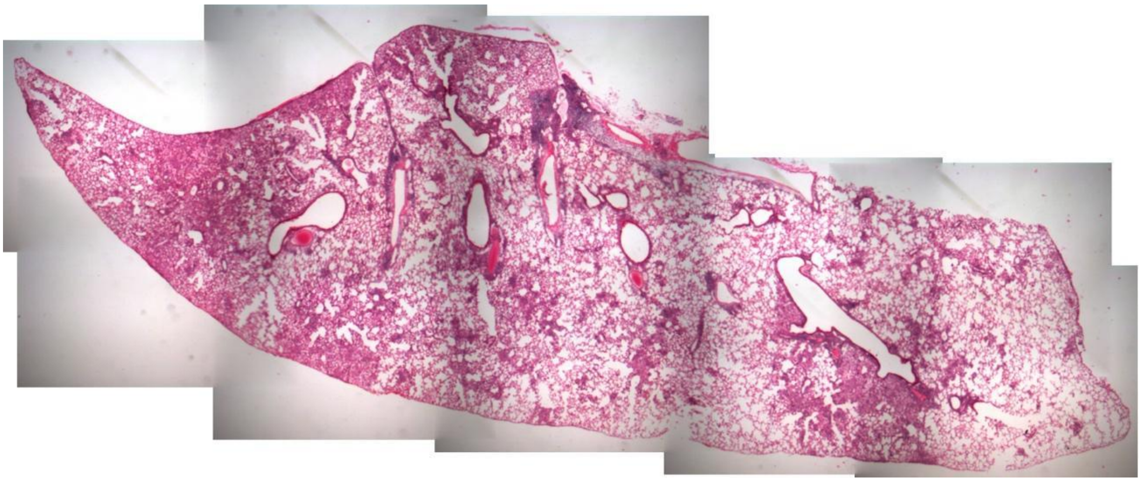

Figure S3. Hematoxylin and eosin (H&E) stained lung tissues BALB/c mice infected with *Mtb*H37RvΔ*ctpA* at 60 dpi.

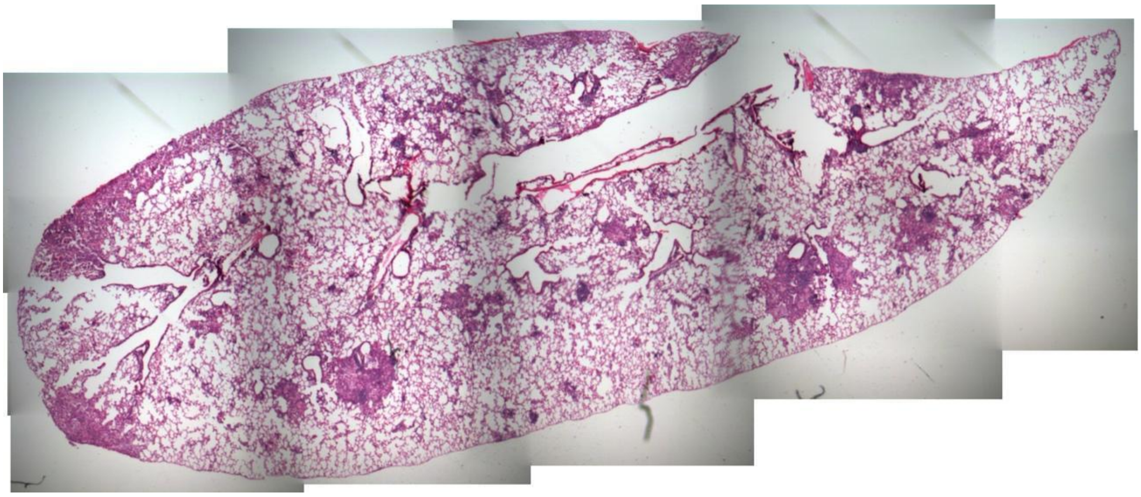

Figure S4. Hematoxylin and eosin (H&E) stained lung tissues BALB/c mice infected with *Mtb*H37RvΔ*ctpA* at 120 dpi.

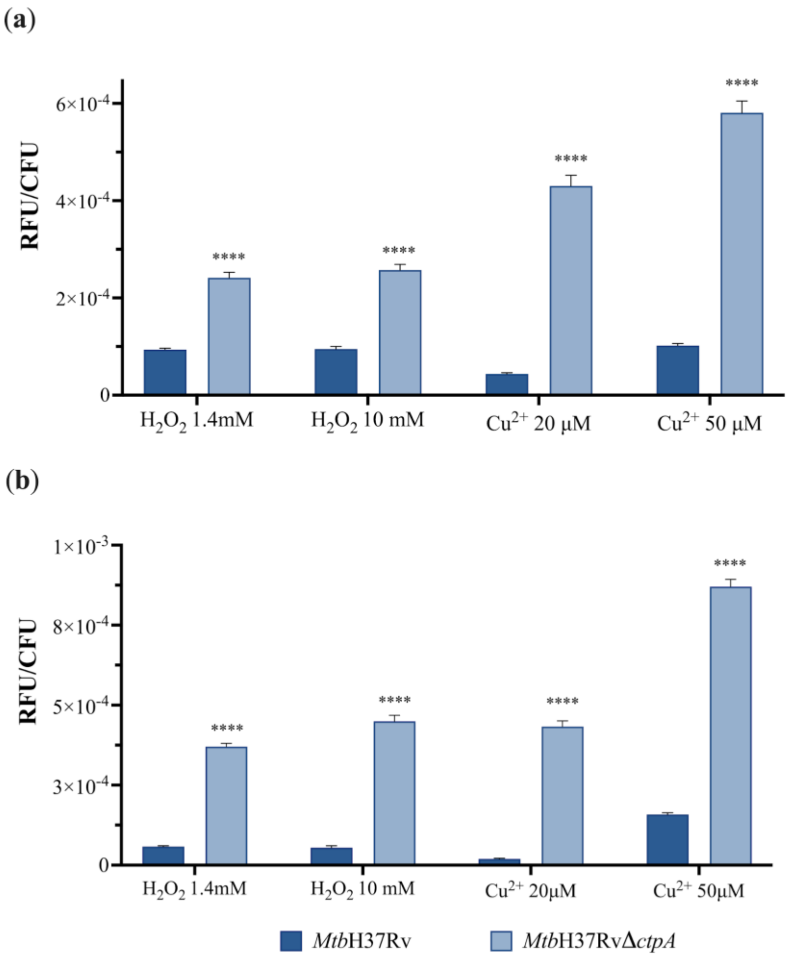

Figure S5. Effect of ctpA deletion on intracellular ROS generation in Mtb under stress conditions. Intracellular quantification of total reactive oxygen species in *Mtb*H37Rv, and *Mtb*Δ*ctpA* treated for 3 h with $CuSO_4$ 20 and 50 μM and H_2O_2 1.4- and 10-mM using fluorescence generated at (**A**) 18 h and (**B**) 36 h by the oxidation of 2',7'-dichlorodihydrofluorescein diacetate (H_2DCFDA) [66]. Asterisks indicate differences in fluorescence levels normalized to CFU relative to the wild-type strain under the same stress condition by two-tailed unpaired t-test (*** $p < 0.001$, **** $p < 0.0001$).
